# Redesign of a Flange Wheel Used in an Airplane for Composite Manufacturing Made with a Mold with Removable Inserts Manufactured by Means of 3D Printing: A Comparison with the Current Conventional Alternative, a Turbine Wheel Machined out of Aluminum

**DOI:** 10.3390/ma18061296

**Published:** 2025-03-15

**Authors:** Carlos Javierre, Víctor Camañes, Julio Vidal, José Antonio Dieste, Angel Fernandez

**Affiliations:** 1 i + aiTIIP Group, Department of Mechanical Engineering, University of Zaragoza, C/María de Luna, 3, 50018 Zaragoza, Aragón, Spain; vcamanes@unizar.es (V.C.); afernan@unizar.es (A.F.); 2 R&D Department, Aitiip Fundation, Empresarium Industrial Site, C/Romero, No. 12, 50720 Zaragoza, Aragón, Spain; julio.vidal@aitiip.com (J.V.); joseantonio.dieste@aitiip.com (J.A.D.)

**Keywords:** thermoplastic parts design, injection processes, removable inserts, 3D printing insert, composite manufacturing, lighter design, turbine wheel

## Abstract

This work presents the redesign of an aircraft aluminum turbine wheel into a thermoplastic composite flange wheel with the support of 3D printing technology, which increases the turbine efficiency thanks to the introduction of the flange geometry, not possible with the current machined aluminum part. This work seeks the reduction of the aircraft’s structural weight by replacing metallic components with thermoplastic alternatives and proves the feasibility of producing a complex geometry product through injection molding, paving the way for manufacturing intricate designs using removable inserts created via 3D printing. This work has been developed within the INN-PAEK project of the H2020-CLEAN SKY 2 program. The thermoplastic component is produced using an innovative process that employs removable inserts in the mold, and its development has followed following three steps: redesign of aluminum part according to functional and plastic materials requirements, design of the mold, and validation of real plastic parts by means of tomography. This paper highlights highly positive results for the project, influenced by the new plastic flange wheel’s ability to achieve both weight reduction and an overall efficiency enhancement that decreases the aircraft’s kerosene consumption, and proves that 3D printing is a highly potential technology for complex thermoplastic part tooling production.

## 1. Introduction

Weight reduction is an important criterion in design processes in various fields. Applying this criterion allows for economic savings, improved functionality, and reduced environmental impact in most cases. In the aeronautical sector, this is a fundamental design guideline. Reducing the weight of an aircraft’s components as much as possible enhances functionality and reduces consumption. Steinegger [1] shows how a reduction of 1 kg of weight saves 0.02 to 0.03 kg of fuel per 1000 km, and Léonard and Nylander [2] highlight how a 100 kg lighter aircraft could save from 124 to 129 tons of fuel over a lifetime. Traditionally, the aeronautical sector has favored lightweight metals such as aluminum and manufacturing processes that, while sacrificing cost, produce the lightest possible parts.

**Figure 1:**
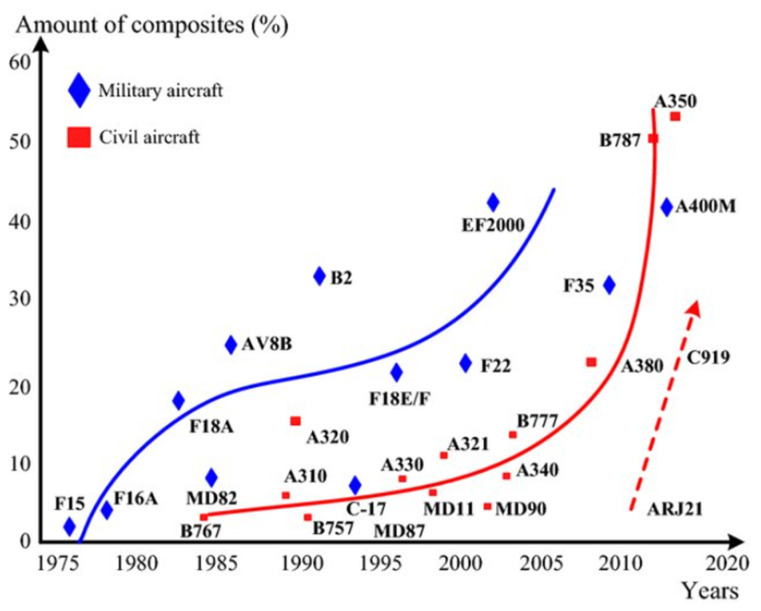
Composite material usage rate evolution during the last decades.

Figure 1 shows the increment of composite material used in aircrafts during the last decades [3]. In this context, we need to add up the current state of thermoplastic composite materials and consider the impact that such materials will have in the industry.

One way to lighten the aircraft is the substitution of metallic components with plastic ones [4,5]. Plastic materials have a lower density than metals, and their lower mechanical properties can be compensated by properly selecting the type of plastic and adding fillers. High-performance composite plastic materials are available on the market and can be used for functions traditionally covered by metals, allowing the design of complex and lighter shapes without increasing costs. Among these materials are thermosets or thermosetting plastics, which have been used in aeronautics for years. These composite materials, reinforced with various types of fiber, can achieve very high mechanical properties and be applied to the structural components of an aircraft. Being thermosets, their manufacturing processes require curing stages and are notably limited in terms of part shapes and cycle times, as the continuous fibers used for these kind of materials, together with the low pressure applied in their production process, makes it difficult for the material to reproduce the complex and small geometries of the mold [6]. They also face significant end-of-life limitations, a factor increasingly considered in design processes.

On the other hand, there are thermoplastic materials, which melt with heat. These materials allow for highly varied manufacturing processes with very short cycle times that depend only on the cooling time of the parts. The market offers a wide range of thermoplastics with very different properties that can also be improved by adding additives and fillers. In the case of parts with very high requirements for dimensional stability, structural rigidity, and operating temperature, reinforcement with fiberglass and/or carbon is preferred to achieve optimal mechanical behavior. The non-continuous fiber composite materials resulting from the mixture of this thermoplastic matrix with non-continuous fiber allow the production of parts with complex geometries in the mold, as these materials can perfectly fill the cavities of these geometries, thanks to the nature of the materials and the high pressures used in the injection process [6].

In addition to their lower density, parts made from thermoplastics can be further lightened using transformation processes specific to these materials, such as foaming, or manufacturing processes that easily achieve thin walls suited to the mechanical demands of the part, like injection molding.

This process is not without some limitations concerning this technology, such as the possibility of droplet satellite appearance and nozzle clogging, among others. The main reason for the appearance of droplets is due to the possible inertial effect of the flow, as happens in steel casting or 3D printing injection, and it is unlikely to occur in the molding of very-high-viscosity materials (up to 1000 Pa·s at 100 1/s shear rate) like PEEK. However, the appearance of the jetting defect is quite likely, which can occur if there is an abrupt change in section from the sprue to the cavity. To solve this problem, it has been decided to inject with a sprue perpendicular to the surface of the cavity to maintain squeeze flow conditions. Additionally, the runner will absorb any droplets that may appear, and due to its thickness, it will not suffer from clogging.

One of the most used processes for manufacturing parts from thermoplastics is injection molding. Injection molding opens the window for complex geometries and therefore increases the production rate and decreases the manufacturing costs. The limitations on the shapes of injected plastic parts mainly arise from the demolding of the piece. Molten material is shaped using a mold, and once it cools down, the mold must be separated from the part. During the design phase, it is always necessary to consider how the mold used to manufacture the part will be, what movements will be performed to open the mold and demold the part, and numerous factors related to separating the part from the different sections of the mold. It is important to think about where the part will grip the most, where it will need to be pushed without damage, the draft angles required for smooth separation, etc. Any incorrect decision on these points can result in defects in the part.

Another very important factor to consider in the design of a part intended to be manufactured from thermoplastic material through the injection molding process is the issue of thicknesses, both in terms of their values and distribution throughout the part. For each material, depending on its viscosity and the flow path required to fill the part, a minimum thickness can be determined to ensure manufacturability. These minimum thicknesses can be less than one millimeter, and many parts are manufactured with thicknesses below that value. To fill molds with such low thicknesses, very high injection pressures are required, which must be considered, especially when designing the mold, to prevent defects in the part. High pressures during the injection process can cause various types of defects related to part quality, internal stresses in the material, and differential shrinkage throughout the part. Additionally, the effects of high pressures on the mold must be carefully considered, including aspects such as mold durability, fatigue on its components, and deformation of the mold sections shaping the part.

Deformation of mold inserts due to rigidity issues is a common problem in molds subjected to very high pressures during the injection process, which results in thickness variations in the part compared to the original design.

Regarding the thicknesses achievable in an injected plastic part, there are also limitations concerning thickness variations throughout the piece. Thicknesses should ideally be as uniform as possible; otherwise, numerous issues may arise during the injection process, leading to defects in the part, if it can even be molded. Defects resulting from non-uniform thickness distribution often include dimensional quality issues, warping, sink marks, voids, and more. Another significant issue is the attempt to achieve very high thicknesses in injected plastic parts, particularly due to the exponential increase in cycle time with part thickness and, consequently, its cost.

If a part must have very high thicknesses or be solid in certain areas to ensure functionality, alternative design options can be considered. For example, the solid portion of the part can be made independently using other technologies and then joined to the rest of the injected piece. One joining method allowed by the injection process is overmolding. In the overmolding process, an insert is first placed in the mold, and then thermoplastic material is injected so that, due to shrinkage or geometry, the two are bonded together. This process eliminates the need for additional assembly processes and allows the joining of parts made from different materials.

As mentioned earlier, the shapes of injected plastic parts can be highly varied without necessarily resulting in a significant cost increase. This versatility in achievable shapes is leveraged in designs to enhance the functionality of parts. In conventional injection molding processes, shape limitations arise mainly from the demolding and ejection systems used in mold fabrication. To reduce shape limitations in injection molds, various mechanisms and technologies have been developed over the years. Slide systems, inclined ejectors, unscrewing mechanisms, or collapsible cores are examples of mechanisms commonly found in molds that allow the manufacture of parts with undercuts that cannot naturally demold with the primary mold opening directions.

A more recently developed option is the use of removable inserts. These inserts are made from materials that are solid but can be dissolved and separated from the part through various systems. They offer the advantage of enabling shapes with undercuts that cannot be demolded using traditional mechanisms. However, they also present certain disadvantages, such as the need to produce inserts for each part, the lower mechanical properties of these materials compared to steel, and slower manufacturing processes due to the need to place the inserts in the mold for each piece.

One of the different options evaluated to generate such hollow structures was the use of salt core technology developed by Ceramtec (Subsidiary Emil Müller GmbH, Plochingen, Germany) [7]. Such components are manufactured by an injection technology, which enables the manufacture of simple geometries. Although they have good thermal and compressive properties, their brittleness and the need of a mold to manufacture can be limiting factors for certain activities.

Within these removable materials for inserts, there are options available on the market that can be used in additive manufacturing systems [8]. An example of such materials is those offered by NEXA3D, which allow the printing of removable inserts for molds.

**Figure 2:**
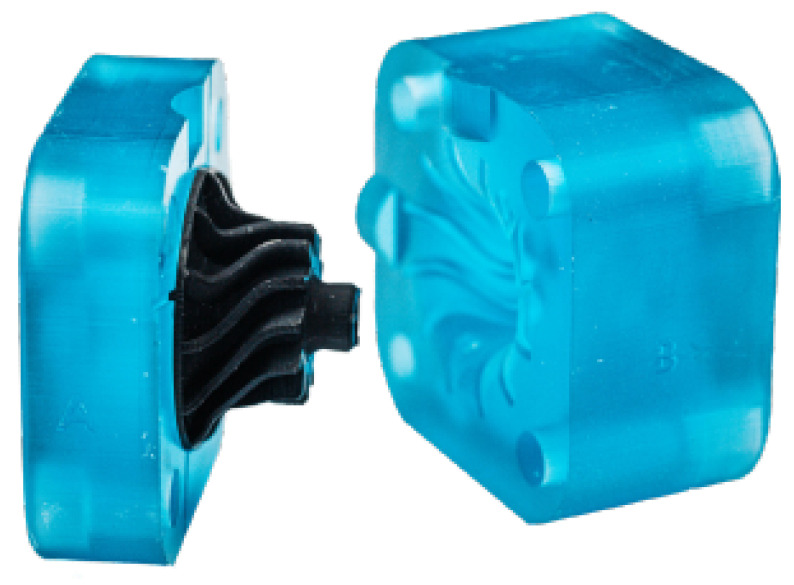
Example of a dissolvable resin mold (xMOLD).

The material called xMOLD is a high-performance dissolvable resin developed for Freeform Injection Molding (FIM), shown in Figure 2, which is manufactured by 3D printing technologies by using resins which are crosslinking under the action different wavelengths (Figure 2). These material manufacturing technologies enable the production of free geometries and allow the generation of a viable solution for complex and hollow parts. The production of the insert varies on time depending on the thickness of the layer, which can go from a minimum of 25 microns to a maximum of 200 microns. To achieve the maximum resolution, inserts were manufactured at a speed of 25 and 100 microns.

Thermoplastic materials encompass a wide range of families with highly varied properties. Generally, their mechanical properties are relatively low compared to metals, but there is a group of highly technical thermoplastics that, through the addition of high-percentage additives, fillers, and reinforcements, significantly improve their mechanical and thermal properties. These materials can sometimes serve as alternatives to certain metals in specific applications. Examples include polyamides, PPS, and PEEK [9]. Such materials are used in components with special mechanical requirements across various sectors, including aerospace, due to their lightweight nature, flexibility in achieving complex shapes, and lower costs. PEEK has shown an incredible mechanical performance on the design of safety-critical aircraft structures, such as a wingbox developed by Clean Sky 2’s [10], proving the potential application of this thermoplastic material, instead of traditional thermoset, simplifying the processing, and allowing weight reduction in aircrafts. As it is possible to add any filler due to its thermoplastic nature, it also offers great possibilities for several applications in the aircraft industry [11].

However, the replacement of metals with reinforced thermoplastics is not without risks, especially if the dimensional tolerances are very narrow. In some aerospace components, the quality and precision of the “wet” surfaces must be very high, because their efficiency will largely depend on them, as is the case with turbines. This efficiency can be optimized if mechanical simulations are performed as [12]. It is essential that the part in operation has almost exact measurements and shapes; therefore, it is important to determine how it will deform when subjected to thermomechanical load and in motion. To carry out more accurate mechanical simulations, it is crucial not to lose sight of new research on methods for characterizing the mechanical properties, such as the Young’s Modulus of substitute materials for metallic alloys [13].

Within the world of additive manufacturing, these advanced technical materials have also been incorporated over time.

Thermosetting materials generally have better mechanical properties, especially in terms of thermal properties. These materials have also been developed for use in additive manufacturing processes.

For example, xPEEK147 [14] was developed with material partner LoctiteAM/Henkel. This high strength and high temperature plastic is suitable for plastic molding tooling. This material is relatively slow-curing and has a thermally cured chemistry, requiring a post-cure bake cycle to achieve advertised properties. This is a strong but brittle material when fully cured. xPEEK147 requires pre-heat to condition the material before use for best results. Heat it in the provided container at 60 °C for 24 h. Let the resin and container cool down to ambient temperature and shake well before dispensing. We recommend heating in a water bath such as a sous-vide apparatus or heated ultrasonic washer with ultrasonics turned off. Keep the container below 65 °C to avoid softening the plastic. Re-heating may be required if not used within 1–2 months. Store the resin in the original plastic container above 20 °C to prevent crystal growth. Dispense through a 190 um filter to check for crystallization. If crystallization occurs, return the material to the container and complete the pre-heat cycle before use.

This work has been developed within the INN-PAEK project of the H2020-CLEAN SKY 2 program. INN-PAEK means innovative thermoplastics transformation processes to produce a flange wheel in PAEK reinforced with short fibers. INN-PAEK is a project focused on increasing the efficiency of manufacturing processes used for aeronautical components, which will also enable and validate the use of new materials and technologies.

## 2. Materials and Methods

The method applied for the redesign of the aluminum wheel in a plastic alternative part is divided into the following three steps:

1. Redesign of the aluminum part to be able to be manufactured with plastic materials.
2. Design of the mold to produce the redesigned part.
3. Validation of real plastic parts by means of tomography.

**Figure 3:**
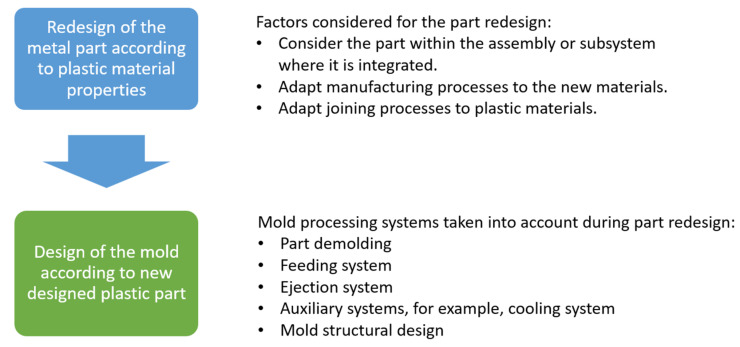
Method overview scheme.

The following picture (Figure 3) shows a scheme of this applied method, explained in detail in the following subsections. 

### 2.1. Redesign of Parts in Plastic Materials

The redesign of a metallic part into plastic is a process that has been carried out in various ways since the appearance of the first plastic materials. This research team has extended experience through years of research on developing metallic part redesign methodologies into plastic, considering such different factors as material properties, processing conditions, or economic criteria, applied in different projects [15,16,17]. As mentioned earlier, the main reasons for this are economic and functional. The extensive range of plastic materials developed, along with the introduction of additives, fillers, and reinforcements, provides such varied properties that they can replace almost any material and be integrated into nearly any sector. Redesigning a component in plastic can be a complex process if the goal is to maximize the benefits of thermoplastics and their manufacturing processes. Optimizing the design and functionality of the part must remain a focus throughout the redesign process. The following methodological factors should be considered when converting a part from a traditional material to plastic:

- Consider redesigning not just the part but also the assembly or subsystem it integrates with: This means avoiding a simple one-to-one substitution of the material without rethinking the design. Instead, the goal should be to maintain the part’s functionality while reshaping it to generate new functionalities that add more value to the redesigned part. The transformation processes for plastic materials enable the creation of complex shapes without increasing costs. These complex shapes can enhance the functionality of parts and even reduce the number of components by combining several elements from the original design into a single plastic part. Achieving a reduction in the number of parts during the redesign process can lower assembly costs, reduce the number of part references, and simplify the mechanical assembly.
- Adapt manufacturing processes to the new materials: Many manufacturing processes applied to traditional materials can also be used for plastics [18]. However, plastics have unique processes that offer numerous advantages. For instance, injection molding in its various forms allows for thermoplastic manufacturing with the following features:
  - Fast and economical production processes.
  - Feasibility for both large and small production runs.
  - Complex shapes without added costs.
  - Variability in aesthetic finishes, surface treatments, and part marking at minimal additional cost.
  - Possibility for post-process operations such as machining, finishing, and painting.
- Adapt joining processes to plastic materials: The joining processes for plastic parts must consider the characteristics of the materials and their manufacturing methods. This includes incorporating joining techniques such as overmolding, adjusting design criteria for traditional methods like screwing, clamping, and riveting, simplifying processes like snap-fits or welding, and, in some cases, complicating others like adhesive bonding. In general, special care must be taken when designing joints to account for the properties of the materials and how they respond to the types of stresses and environments in which they will operate.

### 2.2. Mold Design

A proper mold design is essential for obtaining high-quality parts. The mold design must be developed in parallel with the part design to ensure the part is technically feasible to manufacture and its geometry does not cause defects during production. Various systems within an injection mold need to be developed. These systems are interconnected [19], and if not properly designed, quality parts cannot be produced at all. The main systems within a mold can be grouped as follows:

- Part demolding: When defining the geometry of a part to be manufactured using an injection mold, it is crucial to consider how the part will be demolded and separated from the mold. Some parts naturally demold, but many others have undercuts. For these undercuts, demolding systems must be designed to make manufacturing the part viable. It is also necessary to define the areas requiring draft angles, determine the appropriate draft values, and assess whether these angles impact the part’s functionality.
- Feed system: This system ensures that the mold is filled properly. It influences several factors, such as the mold’s ability to fill, the geometry of the finished part, and its aesthetic and mechanical quality. The design of the feed system affects how material flows within the mold and all related aspects, such as part shrinkage and material orientation.
- Ejection system: As the material cools down inside the mold, it contracts, causing the part to shrink. The mold prevents the part from shrinking in certain dimensions during contraction, leading the part to adhere to specific sections of the mold. Mold design must anticipate where the part will remain attached and include a system to separate the part from the mold in that area. This system will operate after the mold opens and must remove the part without damaging it.
- Auxiliary systems: Various auxiliary systems ensure the mold functions correctly. For example, the cooling system ensures rapid and uniform part cooling. The guidance and centering system ensures that when the mold closes, all its components align correctly to define the part’s geometry. Other auxiliary systems might include elements for mold handling and transportation, parameter recording, and more.
- Mold structural design: The mold is a component subjected to fatigue, undergoing significant pressure, force, and temperature variations in short cycles. For instance, during each cycle, the mold is compressed with the clamping force to prevent it from opening during injection, and the cavity endures extremely high pressures. Thin or large parts made from viscous materials may require injection pressures exceeding 1e8 (Pa). To ensure the mold endures many cycles, the selection of materials, structural element dimensions, plate thicknesses, and component assembly must be carefully designed. Proper joint and alignment designs for all components are also critical.

Injection molding (IM) simulation is an essential technique for the accurate design and development of plastic components [20,21]. It ensures that the mold cavity will be properly filled and that the manufactured part will exhibit acceptable distortion and warpage, as well as thicknesses consistent with the intended design. The execution of simulations is a complex activity that requires significant computational and personnel resources, as any iteration in the design process, such as a modification of dimensions or thicknesses, necessitates redoing the simulations to ensure manufacturability. This is because the physical laws governing the flow within the mold are complex and highly sensitive to variations in both molding parameters and thickness [22,23,24]. In the IM simulations, the continuity, momentum, and energy equations associated with the flow of a non-Newtonian fluid in a volume representing the geometry of the cavity are solved [25,26]. This is discretized using a volumetric mesh. The software used for IM simulations is Cadmould v17.0 (2024). It performs the approximation of the generalized Hele-Shaw flow under non-isothermal conditions. Additionally, it approximates the flow between flat plates, where the distance represents the thickness of the cavity. Furthermore, this is modeled with a flexible 1D element, which significantly reduces computation time and improves calculation predictions [27]. This challenge becomes even more significant when working with complex, thin sections and highly viscous materials such as PEEK filled with a high content of short carbon fiber.

### 2.3. Tomography as a System for Part Verification

Tomography is imaging by sections or sectioning that uses any kind of penetrating wave, allowing the user to see the internal structure of a solid part. The technology is widely used in the medical sector, and it depends highly on the type of material that is going to be studied. Within the project the technique used has been X-ray tomography. Thanks to this technique, it has been possible to check the whole flange wheel in a non-destructive test.

## 3. Case Study

**Figure 4:**
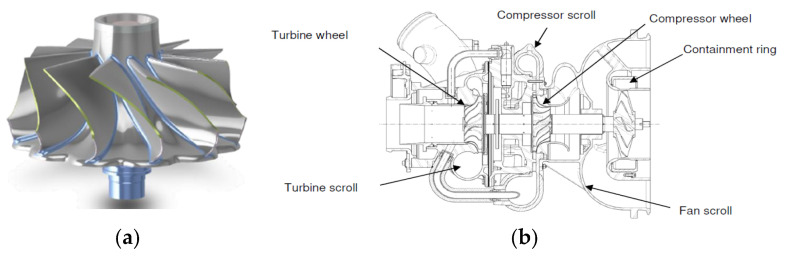
(**a**) Aluminum machined turbine wheel. (**b**) Section of an Air Cycle Machine.

The aim of the present study is to redesign for composite manufacturing a flange wheel used in Pack Air conditioning of an airplane, made in aluminum. The manufacturing and design of the flange wheel in composite material is then compared to the current conventional alternative, a turbine wheel machined out of aluminum, which does not include a flange, which hinders performance (Figure 4). The flange geometry cannot be obtained with a full aluminum turbine wheel, due to its manufacturing process. The objective of this work is not to quantify the difference in efficiency between the current aluminum turbine and the new composite material turbine with the shield component, which is known to improve its efficiency. This evaluation can be performed once the new part is available. Nor will its mechanical behavior be assessed. The mechanical demands on the turbine are not very high; a material commonly used in components with similar requirements in the sector has been selected, and its mechanical behavior can also be evaluated with the new part. Additionally, to make a complete comparison between the designs, a comparative cost analysis could be conducted. In this analysis, real conditions of large-scale manufacturing would need to be considered. The assessment of these costs is not the subject of this article.

### 3.1. Turbine Wheel: Introduction to the Part

The turbine has two zones with very different geometries. The first zone corresponds to the turbine blades, characterized by several curved, thin surfaces distributed along the turbine’s axis of rotation. The second zone, the core area, is a solid, thick section. The turbine shaft is placed within the core.

The manufacturing process for the conventional aluminum part consists of successive machining processes starting from a block of this material. These processes are carried out both to shape the part and to achieve its balance. The aluminum alloy used for the conventional aluminum turbine is a special one, 2618A EAA, used in the aeronautic sector.

The initial version of the part is made from an 1873 g block of aluminum. Through a machining process, 1586 g of material is removed. The final aluminum part, which integrates the core, weights 287 g.

**Figure 5:**
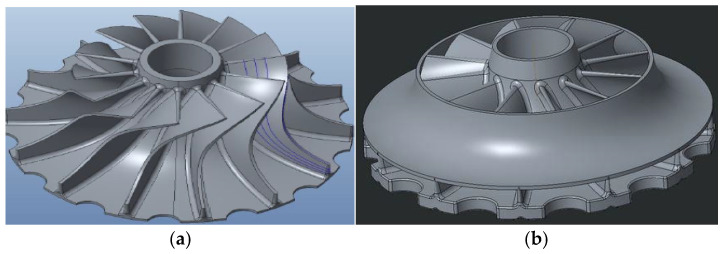
(**a**) Aluminum turbine wheel machined. (**b**) Flange wheel in thermoplastic composite.

The new flange wheel in thermoplastic composite consists of a machined aluminum core, the same aluminum alloy as in the conventional part, and a flange turbine that allows for performance gains. The thermoplastic part (Figure 5) is manufactured with a carbon fiber-reinforced thermoplastic PEEK thanks to a new manufacturing process developed in the INNPAEK project, based on a new removable insert injection molding process, which is explained in the next subsection.

In a second variant of the part, the core is manufactured using additive manufacturing with a thermosetting plastic material very similar to the rest of the piece, called xPEEK147.

### 3.2. INNPAEK Turbine: Redesign of the Turbine for Manufacturing with Removable Insert Molds: Introduction to the Part

This section outlines the redesign process of the turbine to be manufactured from composite plastic material via injection molding. The final geometry of the piece is confidential, but the intermediate geometry, from which the work methodology was developed, is presented.

The turbine redesign could initially have been planned as a simple material replacement, substituting the blades and the core with a single injection-molded plastic piece. However, as explained in the methodology section, this approach would have been incorrect. The proper approach is to adapt the geometry of the injection-molded plastic part to suit the material and its manufacturing processes. In this case, two critical factors were considered: the ability to shape the plastic injection-molded part to enhance and improve functionality and the limitations regarding wall thickness inherent to this type of part.

Regarding wall thickness considerations, injection-molded plastic parts should avoid excessively thick walls or significant thickness variations across the part. Therefore, the decision was made to maintain the core as a separate, independent component rather than integrating it into the plastic part. The union between the core and the injected piece was achieved by overmolding the part onto the core.

The blade section could be manufactured via injection molding without issue, but opportunities for improvement were identified. One significant enhancement was the addition of a shield zone above the blades. This shield increases the turbine’s efficiency and, consequently, the air conditioning system’s overall performance.

In the aluminum version, the shield cannot be integrated due to manufacturing limitations, requiring a separate component and posterior wielding and therefore increasing costs and assembly complexity. A conventional injection-molded plastic part also could not include this feature, as the area between the shield and the blades would create an undercut, preventing demolding with traditional mechanisms.

**Figure 6:**
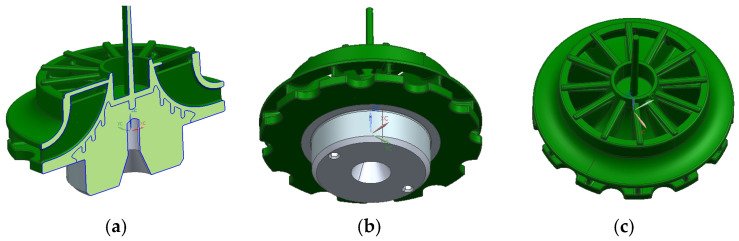
Final turbine geometry. (**a**) Section view. (**b**) Bottom view. (**c**) Top view.

To overcome this, the design incorporated removable inserts that could dissolve and separate from the piece after injection molding, similar to the inserts used in traditional sand-casting processes. This solution enabled the creation of a more efficient part than the aluminum counterpart but introduced a slower and less-typical manufacturing process for injection-molded plastic parts. Figure 6 shows the final geometry of the part.

**Figure 7:**
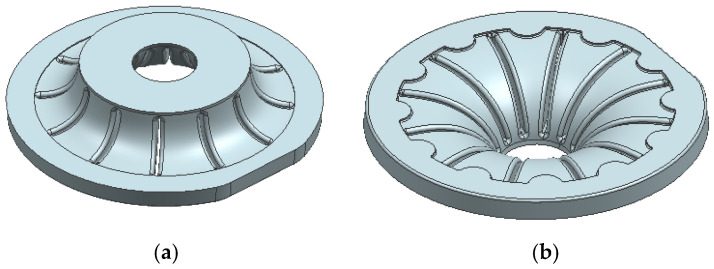
Removable inserts views. (**a**) Top view. (**b**) Bottom view.

The geometry shows a blade area that, at the bottom, would be joined to the solid core, while at the top, it would include the shield area. The lower part would bond with the core due to the contraction of the piece during the injection process, as the core would be placed into the mold prior to injection. The area between the blades would remain undercut, making demolding with conventional mechanisms impossible and requiring the use of removable inserts. This approach would allow for almost any blade and shield geometry to be achieved, maximizing efficiency. The following figures (Figure 7) show an initial version of how the removable inserts might shape the interior of the piece. Single-piece inserts are proposed to ensure maximum rigidity and avoid issues with insufficient stiffness that could arise from multiple individual inserts needing to be held together.

**Figure 8:**
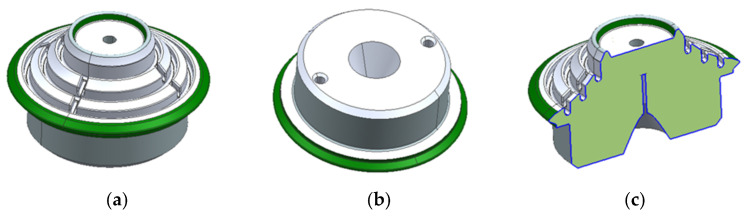
Core insert part. (**a**) Top view. (**b**) Bottom view. (**c**) Section view.

In addition to the removable inserts in the mold, the core which provides the solid section of the thick piece must be inserted prior to injection. Figure 8 shows the geometry of this core.

**Figure 9:**
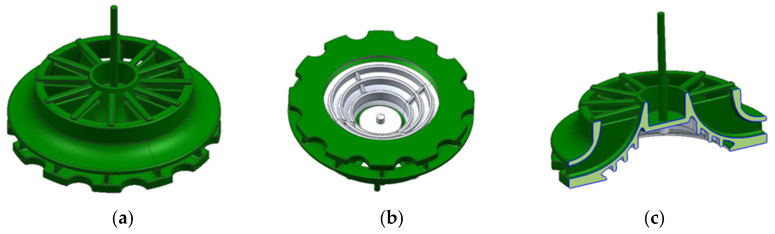
Injected part model. (**a**) Top view. (**b**) Bottom view. (**c**) Section view.

Finally, it would be necessary to define the blade area with the shield and the section in contact with the core. In Figure 9, the geometry of the piece to be produced by injection is displayed. The shield section, where the material would be fed into the mold, is visible. Since the shield is a revolution piece, a central feeding system extending radially to the shield was deemed appropriate. This arrangement enhances material orientation and therefore reduces piece contraction. The lower part includes radial and circular ribs designed to improve bonding with the removable core during overmolding.

**Figure 10:**
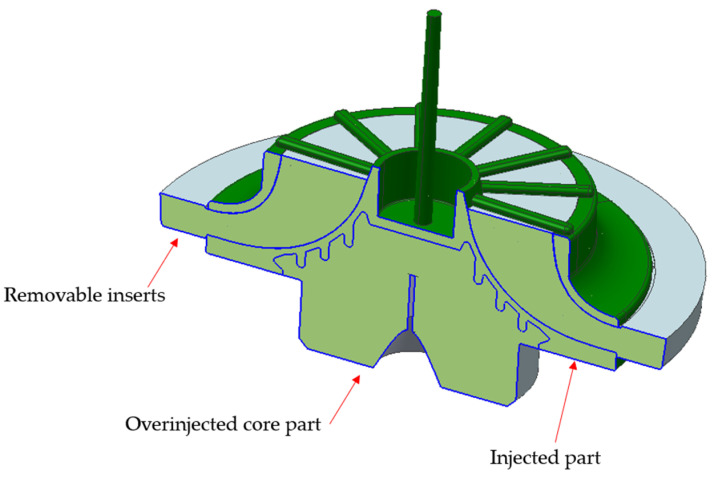
Final injected part section, showing removable inserts, core part, and injected part.

After the injection process, the part would consist of the overmolded core and the removable inserts inside, which would later be removed to obtain the final piece (Figure 10).

The total weight of the part developed in the INNPAEK project is 265.2 g, of which 179 g corresponds to the aluminum insert and 86.2 g to the composite material. Of the 86.2 g of the plastic part, 27.88 g corresponds to the shield component.

### 3.3. INNPAEK Turbine Manufacturing and Validation of Manufacturing Process

The manufacturing process of the turbine in the INNPAEK project has three stages. First, the aluminum core must be machined from a block of this material. Second, the inserts must be manufactured either through molding or 3D printing. Finally, the overmolding process of the aluminum core must be carried out using the mold where the soluble inserts have been placed.

**Figure 11:**
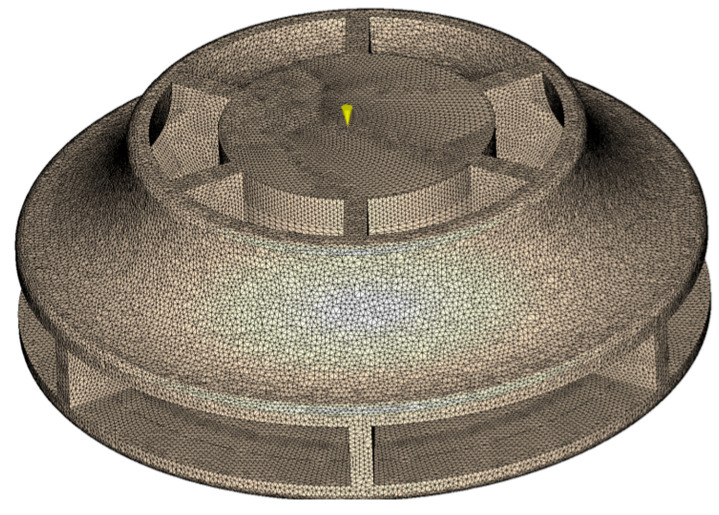
Finite element mesh of the part.

To validate the proper design of the part and mold regarding the injection process, several simulations were performed, varying the process conditions until they were optimized. These optimum parameters for mold filling, post-filling, cooling, and warping are shown in Table 1. Optimization was made under the criteria of minimum flow front temperature decrease, filling pressure not exceeding 800 bar, and minimum volumetric shrinkage variation around the cavity. All these criteria ensure the material consolidation after solidification is good enough to achieve mechanical properties as warpage is reduced. From the 3D geometry of the part, a mesh like the one shown in Figure 11 is generated.

The material selected for the simulation is a PEEK filled with 30% short carbon fiber. The grade is Victrex 450CA30, supplied by Victrex. The main properties are obtained from the Cadmould database (Table 1). The Carreau–WLF model is applied to determine the influence of temperature and shear rate on melt viscosity [28,29,30]. The IKV–Schmidt model is used to determine the influence of temperature and pressure on specific volume [31].

**Figure 12:**
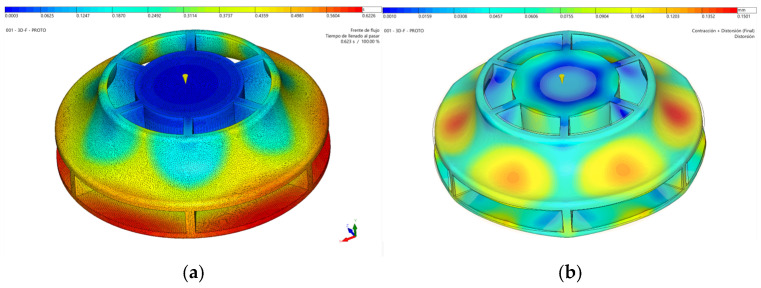
Injection simulation results. (**a**) Flow front advance simulation. (**b**) Part distortion simulation.

The simulations demonstrate how the mold will be filled, as shown in Figure 12a. This outcome is critical for several factors. It ensures the part is filled and advances uniformly within the mold, avoiding areas where material flow could slow or accelerate excessively, which would affect the surface quality. Filling also determines the material and fiber orientation, influencing the mechanical behavior and contraction of the part. This orientation can also be observed as a simulation result. The flow direction, shown as black arrows, induces a high degree of orientation, causing anisotropy.

As previously mentioned, being a circular piece filled from the center aids in uniform contraction and minimizes deformation. Figure 12b shows a maximum distortion in the part below 0.4 mm. Distortion measures the difference between the injected and designed parts. The injected part shows a slight loss of flatness at the entrance of the impeller of 0.12 mm. Also, a loss of circularity at the exit appears due the presence of twelve blades that act as ribs, preventing the contraction of this area in the radial direction. Although the anisotropy induced in the material can aggravate this problem, simulations show that a distortion of 0.045 mm is not exceeded. A distortion at the exit of the impeller of less than 0.1% is considered admissible for the correct hydrodynamics of the turbine.

Another crucial aspect when analyzing filling is assessing how the material advances on both sides of the removable cores to minimize their deformation and ensure correct part thicknesses. Regarding this point, it is essential to monitor the pressure distribution results within the cavity; significant differences between opposite surfaces of the cores could lead to deformations in these components.

**Figure 13:**
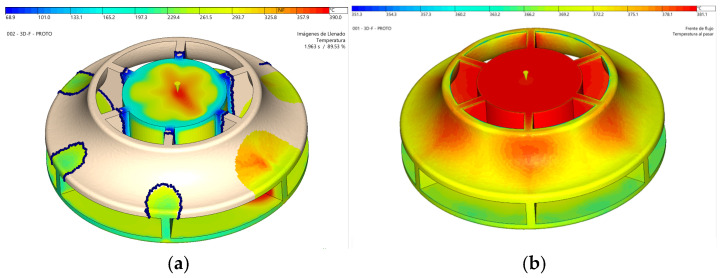
Temperature distribution simulation. (**a**) Unfilled cavity. (**b**) Filled cavity.

One of the most serious problems that can arise when injecting very high viscosity materials like PEEK to make parts with dissimilar and small thicknesses is the appearance of the hesitation effect. This causes a slowdown in the flow in the thin areas; therefore, the flow front temperature decreases, and the cavity may not be filled. A simulation including insert deformation has been developed to study the case of salt or polymeric inserts. These kinds of inserts lack stiffness compared with metallic ones. The inserts of this mold will comprise and bend tenths of a millimeter due to the high cavity pressure and the inlet location. Flow hesitation of high viscosity PEEK material will increase dramatically, and flow front will freeze so that cavity filling should be incomplete. The mold was designed without cooling in the removable insert area, as new inserts must be placed in the mold for each cycle. For this reason, inserts cannot be heated over 140 °C, as recommended for PEEK injection molding, so that this short shot definitively appears. Figure 13a shows the temperature when 89% of the cavity has been filled, and it is observed that the luxury front is solidified since it has dropped below the “no flow temperature”, which is 335 °C. An optimization of the cavity thicknesses has been developed, reducing the dissimilarities to ensure the balanced filling of the inserts and the complete filling of the cavity. Also, process conditions have been optimized to reduce flow hesitation. Figure 13b shows a complete cavity filled and how the flow front temperature does not decrease below 351.3 °C.

## 4. Results and Discussion

The project results show the parts obtained using the different types of developed inserts. The part was injected using the same mold structure, which will also be presented in the following sections. First, we compare the injection with salt and 3D printed removable inserts, and second, we compare the core part overinjection with aluminum and 3D-printed plastic for this core part.

### 4.1. Mold with Salt and 3D-Printed Inserts

**Figure 14:**
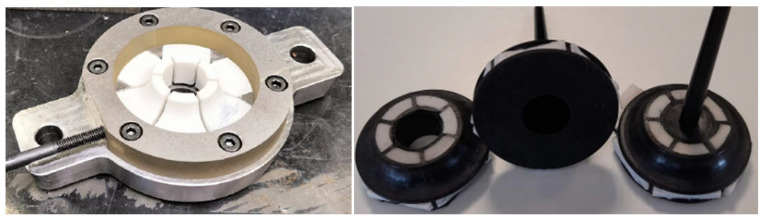
Salt removable inserts and injected samples.

In its initial version, the inserts were made of salt using a molding system. This system allowed the production of small inserts that were individually assembled into the mold. The following images (Figure 14) show the inserts placed in the mold’s registration plates before injection and, subsequently, the injected part without removing the inserts.

During these initial tests, it was observed that the inserts experienced slight movements during injection, resulting in part thicknesses differing from the expected theoretical values.

**Figure 15:**
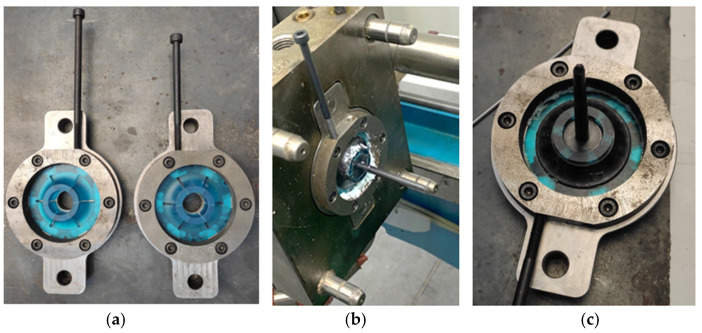
xMold 3D printed inserts. (**a**) Inserts in the mold cavity. (**b**) Injected art in the mold. (**c**) Detail view of the injected part with the inserts.

In a second test, the inserts were produced using 3D printing with a material called xMold, developed by NEXA3. This additive manufacturing system provided greater flexibility in shaping the inserts and, consequently, in shaping the part. Additionally, the inserts could be made as a single piece, eliminating the loss of rigidity caused by joints between separate inserts. This resulted in a much more rigid design. Figure 15 shows the inserts in their mold housing and the injected part.

### 4.2. Part with Core Manufactured by 3D Printing vs. Aluminum

**Figure 16:**
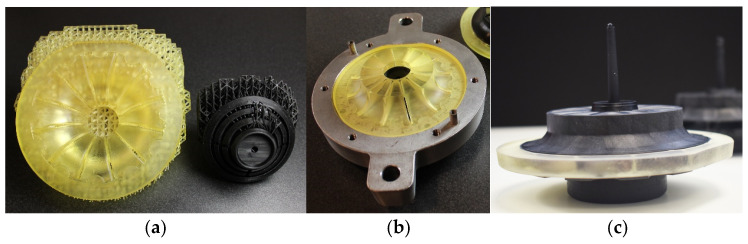
Overinjected samples. (**a**) Removable inserts (yellow) and 3D-printed core part (black). (**b**) Assembled mold with inserts and core part ready for injection. (**c**) Final overinjected part.

From this design, using 3D-printed inserts, a variable was considered to simplify the manufacturing process and further lighten the part acting on the core. This involved replacing the aluminum core with a 3D-printed core. Using 3D printing, thicker sections that are unsuitable for the injection process may be obtained, so this property matched perfectly to produce the solid core part with plastic. Figure 16 shows the result of overinjecting the wheel over the 3D-printed core part, both black colored, using 3D removable inserts, which corresponds to the yellow part. In this final case, all inserts are 3D-printed.

**Figure 17:**
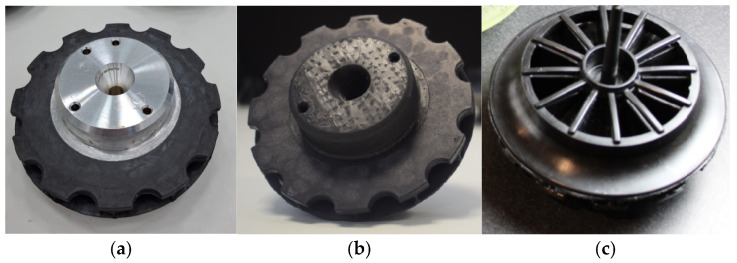
Final samples. (**a**) Aluminum overinjected core detail view. (**b**) 3D-printed overinjected core detail view. (**c**) Top view of the injected part (common to both core part alternatives).

Figure 17 shows the final parts, both with aluminum and 3D-printed core part, once the yellow inserts are removed.

### 4.3. Geometrical Analysis of the Part

To ensure the piece’s geometry, analyses were conducted on the obtained parts. Some were sectioned to examine hidden areas and the material’s interior. A tomographic analysis was also carried out.

These trials have been done just with the 3D-printed thermoset cores. That enables to observe the flow of the material. Nevertheless, the behavior of this material is not the same as the one expected for the aluminum part. It allows the study and understanding of the injection molding process as well as the behavior of the injected part.

It is important to clarify that the part with the insert core, made via 3D printing using a thermosetting material, was not a functional part of the intended application. The aluminum core plays a critical role in the mechanism where the heat evacuation turbine is mounted, a function that cannot be fulfilled with a plastic material due to its low thermal conductivity. The purpose of this test was to demonstrate that in other applications without heat evacuation requirements, the overmolding of thick parts made through 3D printing is a technically viable solution with numerous potential advantages. In such cases, the weight of the overmolded plastic core would be approximately half that of the aluminum part, and greater flexibility in design would be achieved when defining its geometry.

As the external part of the flange wheel can be observed directly after the injection molding process and the blades, quality can be also observed after the solution of the removable inserts. The inner part and stage of the thermoplastic material in direct contact with the core must be studied at the first stage by cutting the wheel in several parts and observing the difference between the parts. In these cases, it is possible to see that the thermoplastic material is completely filling the wheel and achieving a good-quality part. Nevertheless, failures can be observed through the core. As all these failure points are placed in those areas in which a small thickness of the core material can be found, they are related with the brittleness of the material. In this sense, it is focusing its efforts in the identification of possible air bubbles created by the injection molding process. In this sense, it was impossible to identify bubbles (characteristized by the burnting that they generate), and therefore, it was decided to move forward with the quality characterization of the part.

**Figure 18:**
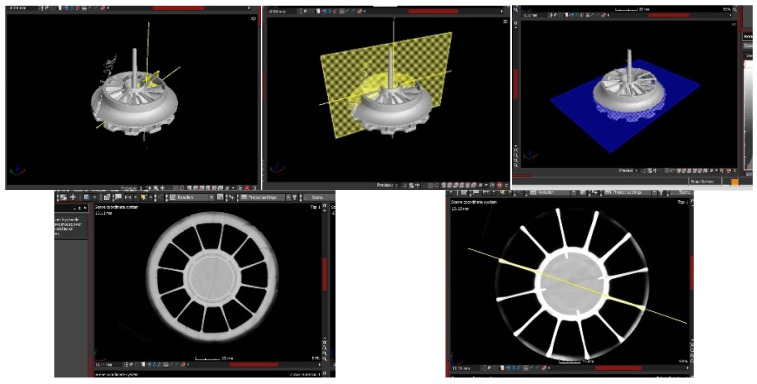
Flange wheel tomography (PEEK 40%CF).

Within the INNPAEK project, the technique used has been X-ray tomography. Thanks to this technique, it has been possible to check the whole flange wheel in a non-destructive test. Through tomographic analysis, the geometry of the final part and the distribution of actual thicknesses in the piece were verified. The results presented in this article are subject to the confidentiality of the turbine’s design in the blade area. Some screenshots of the first tomography done to the flange wheel made of CF can be seen in the following pictures (Figure 18).

The main positive aspect of the new part is the weight reduction, especially relevant for the aeronautic sector, where a weight reduction in aircrafts brings a higher efficiency, which results in lower fuel consumptions and emissions of the airplane. The flange wheel of thermoplastic composite weighs less than the current aluminum wheel, as shown in Table 2.

Therefore, direct savings due to the lower weight transported in the airplane need to be considered. The conventional aluminum wheel has a weight of 0.287 kg versus the 0.2642 kg of the composite part from the INNPAEK project. This 22.8 g makes a difference in fuel consumption during the whole lifetime of the part and during all those flown kilometers. If we compare it with Option 2, the weight saving will be of 108 g.

On the other hand, the new design, thanks to the more-complex geometry due to the flange, improves the overall efficiency of the Air Cycle Machine and, therefore, a lower kerosene consumption of the airplanes and consequently the CO_2_ emissions created.

## 5. Conclusions

In the INNPAEK project, a process to manufacture a thermoplastic flange wheel has been developed. This thermoplastic material, PEEK CF40, filled with carbon fibers, has very high performance, and injection molding is used to manufacture the component. The geometry of the flange turbine wheel is very complex, and it is not possible to obtain it by means of machining, nor by conventional injection molding. To accomplish the geometry, the following manufacturing techniques have been applied:

1. Additive manufacturing technology has been applied for the redesign of an aluminum part into a plastic one. On the one hand, additive technology was used to produce removable inserts for complex cavities production, and on the other hand, to produce a thick core part to be overinjected, made of plastic, optimizing its weight compared with the aluminum solution.
2. An injection mold was produced, using 3D-printed removable inserts, in combination with a core part to be overinjected, producing plastic wheels both with aluminum and 3D-printed cores.
3. Lighter parts were obtained, thanks on one side to the redesign of the part with plastic, possible due to the 3D-printed removable inserts, and on the other side, thanks to the use of additive manufacturing technology to the production of the core part in plastic, lighter than the aluminum core and only feasible to be produced in plastic with this kind of printing technology.

In addition, thanks to this process, the geometry of the new wheel in thermoplastic composite includes a flange, which improves the overall efficiency, only suitable to be done thanks to the removable inserts concept.

This report shows overall very positive results for the project. As has been already explained, the results are influenced by the fact that the INNPAEK flange wheel achieves both weight savings and, especially, an overall efficiency improvement that reduces the kerosene consumption of the plane.

This new technology is aligned with new plastic technology future research lines, where thermoplastics will be used in the aeronautical sector to reduce weight and improve the recyclability of the components, in comparison with current thermoset composite materials [32,33].

## Figures and Tables

**Table 1:** Injection process parameters.

Result	Value
Optimized melt temperature (°C)	380
Optimized mold temperature (°C)	190
Optimized post-filling pressure (Bar)	700
Optimized filling time (s)	0.62
Optimized post-filling time (s)	3.3
Optimized cooling time (s)	7.1
Optimized cycle time (s)	28.4
Max. required pressure (Bar)	612.5
Min. flow front temp. (°C)	351.3
Max. shear stress (MPa)	2.0
Volumetric shrinkage variation (%)	3.6

**Table 2:** Weight comparison of original turbine and two analyzed alternatives.

	Original		New Turbine Option 1		New Turbine Option 2	
Part	Material	Weight (g)	Material	Weight (g)	Material	Weight (g)
Flange Wheel	Aluminum	287	PEEK	86.2	PEEK	86.2
Core			Aluminum	179	X-PEEK	92.8
Total		287		264.2		179

## Data Availability

The data presented in this study are available on request from the corresponding author due to confidentiality.
